# Distinct transcriptomic features of tumor and stromal cells in direct contact in luminal and triple-negative breast cancers

**DOI:** 10.37349/etat.2026.1002388

**Published:** 2026-07-29

**Authors:** Anna Yu. Kalinchuk, Ivan A. Patskan, Evgeniya S. Grigorieva, Liubov A. Tashireva

**Affiliations:** Fondazione Policlinico Universitario Agostino Gemelli IRCCS, Italy; The Laboratory of Molecular Therapy of Cancer, Cancer Research Institute, Tomsk National Research Medical Center, Russian Academy of Sciences, 634050 Tomsk, Russia

**Keywords:** luminal breast cancer, triple-negative breast cancer, spatial transcriptomics, Visium 10x, tumor cells, tumor microenvironment, ligand-receptor pairs

## Abstract

**Aim:**

The aim was to determine the transcriptomic features of tumor and stromal cells in direct contact within tumor nodules in luminal and triple-negative breast cancer.

**Methods:**

Spatial transcriptomic profiling was performed using the Visium 10x platform on FFPE tumor tissue sections from 10 patients with luminal breast cancer and 9 patients with triple-negative breast cancer. Manual morphological annotation of spots and evaluation of differentially expressed genes (DEGs) in identified spot clusters were performed using Loupe Browser v8.0.0 (10X Genomics, USA). Activated biological processes were assessed using the Enrichr online resource and the GO Biological Process 2025 database. Ligand-receptor pairs were identified using the CellChat package (v2.0) in R (v4.4.2).

**Results:**

In luminal breast cancer, mixed cluster (tumor cells colocalized with stromal cells) was characterized by overexpression of genes encoding S100A family Ca^2+^-binding proteins (*S100A4*, *S100A8*, *S100A9*), matrix metalloproteinases (*MMP2*, *MMP7*, *MMP14*), cytokeratins (*KRT5*, *KRT7*, *KRT15*, *KRT23, KRT81*), the mesenchymal marker *VIM*, and epithelial-mesenchymal transition (EMT)-associated genes (*ICAM1*, *PRRX1*) compared to tumor-only cluster. In triple-negative breast cancer, mixed cluster showed overexpression of *S100A2*, *S100A8*, *S100A9*, the epithelial gene *KRT6B*, the cancer stem cell marker *CD44*, and *NOTCH2*, which is associated with negative regulation of EMT. In both breast cancer subtypes, mixed cluster showed transcriptomic enrichment of gene sets associated with regulation of the ERK/MAPK cascade, apoptosis, and cell adhesion and migration. Ligand-receptor pairs associated with cell-cell contact, EMT, and immune response were also detected in colocalized cells, with a broader spectrum of these pairs observed in luminal breast cancer.

**Conclusions:**

This study assessed the transcriptomic characteristics of directly contacting tumor and stromal cells and identified the spectrum of ligand-receptor pairs mediating their interactions. Characterizing the properties of cells at the tumor-stroma interface helps unravel mechanisms of breast cancer progression and identify novel diagnostic markers and therapeutic targets.

## Introduction

Breast cancer (BC) is a heterogeneous disease in which prognosis and treatment response are influenced by both the intrinsic properties of tumor cells and their surrounding microenvironment. The molecular subtype of the tumor currently plays a fundamental role in guiding treatment decisions. Among the various subtypes, luminal and triple-negative BC are the most diverse in terms of clinical behavior and biological characteristics.

Luminal BC is hormone receptor-positive (HR^+^) and is further classified into two distinct subtypes: luminal A and luminal B. Luminal A tumors are characterized by estrogen receptor (ER) and progesterone receptor (PR) positivity with low Ki-67 expression (< 20%), reflecting low tumor proliferation. In contrast, luminal B tumors also express ER and PR but present with higher Ki-67 levels and can be either HER2-positive or HER2-negative (human epidermal growth factor receptor 2). Luminal BC is the most frequently diagnosed subtype and is typically the least clinically aggressive [1]. At the molecular level, luminal A tumors show increased expression of genes linked to ER function, including *BCL2*, *ESR1*, *PGR*, and *FOXA1*. Luminal B tumors share elevated expression of *BCL2*, *FOXA1*, *CCND1*, and *GATA3* but differ by expressing higher levels of proliferation-associated genes such as *CCNB1*, *CCND1*, *CCNE1*, *MYBL2*, and *MKI67* [2]. Notably, tumors of the luminal subtype are generally immunologically “cold,” characterized by low infiltration of immune cells, particularly tumor-infiltrating lymphocytes (TILs). However, when high TIL levels are present, they have been associated with adverse prognostic features, including high histological grade and lymphovascular invasion [3].

Triple-negative BC is a clinically aggressive subtype accounting for approximately 10% of BC cases. It is defined by the absence of ER, PR, and HER2. Triple-negative BC typically exhibits a high Ki-67 proliferation index (> 30%) and is associated with the poorest prognosis among BC subtypes [1]. Basal-like BC, which is often over-lapping with triple-negative BC, is characterized by increased expression of basal cytokeratins 5, 14, and 17, along with dysregulation of key signaling pathways including PI3K/AKT, JAK/STAT, and ERK/MAP. These tumors also show high expression of proliferation-associated genes such as *FOXM1*, *c-MYC*, *CCNE1*, *BIRC5*, and *CCND1*. Additionally, there is elevated expression of genes involved in cell cycle regulation (*CDC20*, *CDC6*) and the epidermal growth factor receptor (EGFR) pathway, coupled with decreased expression of estrogen-related genes [2]. Notably, high levels of TILs are more frequently observed in triple-negative BC compared to luminal subtypes. The presence of abundant TILs in triple-negative BC is associated with a more favorable prognosis and improved response to neoadjuvant chemotherapy [3].

The list of approaches for BC research that hold potential for translation into clinical practice is expanding [4], making it possible to move beyond the study of morphological tumor characteristics and toward an emphasis on the functional state of the tumor and its microenvironment, including intercellular contacts. Recent advances in single-cell RNA sequencing (scRNA-seq) and spatial transcriptomics have enabled the development of detailed molecular atlases that capture the heterogeneity of both tumor and stromal cell populations in BC [5, 6]. While the characteristic features of tumors and their microenvironments across different BC subtypes are increasingly well understood, data on tumor-stromal interactions specific to these subtypes remain scarce. This gap is significant given that interactions between tumor and microenvironmental cells are recognized as key factors influencing chemotherapy response, recurrence, and metastasis [7, 8]. Given that luminal and triple-negative BCs exhibit fundamentally different clinical behaviors, treatment responses, and microenvironmental compositions, the transcriptomic features of tumor cells and their adjacent stromal partners may be subtype-specific. Defining these distinct features is a prerequisite for developing subtype-informed spatial biomarkers that could refine diagnostic classification or predict differential therapeutic sensitivity.

The aim of our study was to investigate the transcriptomic features of tumor and stromal cells in close proximity within tumor nodules of luminal and triple-negative BC. We employed Visium 10x spatial transcriptomics profiling, which enables the integration of transcriptome data with tumor tissue morphology, allowing detailed molecular characterization of cell populations in specific morphological regions. Special emphasis was placed on the precise manual annotation of these tumor tissue regions. We identified three key zones: regions containing isolated tumor cells (tumor-only cluster), regions with isolated stromal cells (stromal cluster), and regions where tumor and stromal cells are in direct contact (mixed cluster). For patients with luminal and triple-negative BC, we generated gene expression profiles specific to the contact regions, identified the most active biological processes, and pinpointed characteristic ligand-receptor pairs that may mediate intercellular communication.

## Materials and methods

### Patients and tumor samples

The study included formalin-fixed, paraffin-embedded (FFPE) tumor tissue samples from 19 BC patients treated at the Cancer Research Institute, Tomsk National Research Medical Center. The cohort consisted of invasive carcinoma of no special type (NST) at stages I–IV and grades 2–3, including 10 patients with luminal A and B subtypes and 9 patients with triple-negative BC (Table 1, Table S1). Tumor samples were collected prior to neoadjuvant chemotherapy. The study was conducted in accordance with the Declaration of Helsinki and received approval from the local ethics committee (protocol no. 4, dated March 3, 2025). Informed consent was obtained from all participants.

**Table 1:** Clinicopathological characteristics of patients.

**Parameter**		**Patients with luminal BC (*n* = 10), % (abs)**	**Patients with triple-negative BC (*n* = 9), % (abs)**
Age*, years		47.5 (34.75–57)	40 (33–60.5)
T	1	50 (5)	44.4 (4)
	2	40 (4)	11.1 (1)
	3	0 (0)	11.1 (1)
	4	10 (1)	33.3 (3)
N	0	50 (5)	55.6 (5)
	1	30 (3)	33.3 (3)
	2	10 (1)	11.1 (1)
	3	10 (1)	0 (0)
M	0	80 (8)	100 (9)
	1	20 (2)	0 (0)
Grade	1	0 (0)	0 (0)
	2	100 (10)	77.8 (7)
	3	0 (0)	22.2 (2)
Ki-67	< 20%	40 (4)	22.2 (2)
	≥ 20%	60 (6)	77.8 (7)
Neoadjuvant chemotherapy		30 (3)	55.6 (5)

*: Age is presented as median (interquartile range). BC: breast cancer; M: metastasis; N: nodes; T: tumor.

### Determination of breast cancer molecular subtype

To determine the molecular subtype of the tumor, immunohistochemical staining for ER, PR, HER2, and Ki-67 proteins was performed. The hormone receptor status (ER and PR expression) was evaluated using the Allred scoring system [9]. HER2 status was assessed according to the ASCO/CAP guidelines [10]. Ki-67 expression was evaluated following the methodology recommended by the International Ki67 in Breast Cancer Working Group [11].

### Spatial transcriptome profiling

For spatial transcriptome profiling, the Visium 10x technology was employed. All material preparation steps adhered strictly to the Visium Spatial Gene Expression Reagent Kits for FFPE protocol. Detailed descriptions of histological sample preparation, library construction for sequencing, and the RNA sequencing process using the 10x Visium technology are available in the original publications [12, 13].

### Bioinformatics processing of raw spatial transcriptomics data

Raw sequencing data (FASTQ files) generated by the 10X Genomics Visium platform were processed using Space Ranger v1.3 (10X Genomics, USA) with default parameters, aligning reads to the human reference genome GRCh38. Manual annotation of tissue sections was performed using Loupe Browser v8.0.0 (10X Genomics, USA), with the detailed annotation methodology described separately. Downstream analyses were conducted in R (v4.4.2) using the Seurat package (v5.0.0) [14]. Data preprocessing included filtering spots with thresholds of nCount_Spatial > 200 and nFeature_Spatial > 15. Normalization and identification of highly variable genes were carried out using the SCTransform method with default parameters [15]. Dimensionality reduction and visualization were performed using principal component analysis (PCA) with the first 30 components, followed by Uniform Manifold Approximation and Projection (UMAP) with default settings. Integration of data from 19 tissue sections and correction for batch effects were achieved using the Harmony package (v0.1.1) [16].

### Identification of differentially expressed genes

Differential gene expression analysis was performed in Loupe Browser v8.0.0 (10x Genomics, USA) using the *Across Multiple Samples* option. In this mode, UMI counts are first aggregated at the level of pseudobulk profiles (by sample and spot class), and the statistical test is then applied to these aggregated profiles rather than to individual spots. Gene expression levels were compared between mixed spots (regions with colocalization of tumor and stromal cells) and stromal-only spots, as well as between mixed spots and tumor-only spots, separately in luminal and triple-negative BC patients. For each gene, we computed average expression levels (normalized mean UMI counts in the selected pseudobulk profiles), log2 fold changes (log2FC, base-2 logarithm of the ratio of normalized mean UMI counts between groups), and adjusted *p*-values (*p*_adj, Benjamini–Hochberg correction for multiple hypothesis testing) using the Wilcoxon rank-sum test. Gene lists were ranked by the absolute value of the log2 fold change, |log2FC| (from highest to lowest). Differentially expressed genes (DEGs) were defined as those with an adjusted *p*-value < 0.05 and |log2FC| > 0.58. *p*_adj = 0.0000 indicates that in the output, *p*_adj < 0.0001.

### Functional enrichment analysis and ranking of activated biological processes

Biological process enrichment analysis was conducted using the Enrichr web tool [17]. Complete sets of upregulated genes (adjusted *p*-value < 0.05, log2 fold change > 0.58) from each subgroup were analyzed against the Gene Ontology Biological Process 2025 database. Resulting biological processes were ranked according to adjusted *p*-value significance (from lowest to highest), with enriched processes defined by an adjusted *p*-value < 0.05. To improve interpretability and reduce the influence of individual terms with borderline significance, similar enriched GO terms were grouped into broader functional categories.

### Ligand-receptor interaction analysis

Cell-cell communication analysis was carried out in R (v4.4.2) using the CellChat package (v1.5.0) [18]. The input data comprised spatial transcriptomic profiles (Visium, 10x Genomics) from 10 tissue sections of luminal BC patients and 9 sections from triple-negative BC patients. Normalized expression data from all samples within each clinical subgroup were merged into a single matrix, and spatial coordinates of all spots were extracted and converted to micrometers.

A CellChat object was constructed in spatial mode (datatype = "spatial") using the standard human ligand-receptor interaction database (CellChatDB.human), which encompasses both secreted and contact-dependent interactions. Following the CellChat v2.0 protocol for spatial transcriptomics, ligand–receptor interaction probabilities were calculated using a truncated mean estimator of group-level expression (type = "truncatedMean", trim = 0.1) to reduce the influence of outliers and low-level noise. Spatial constraints were imposed by specifying a maximum interaction distance of 250 μm between spot centers for the overall search space, and an additional, more stringent threshold of 100 μm was applied to define contact-dependent interactions corresponding to approximately one to two Visium spot diameters. Statistical significance of interaction probability estimates was assessed via bootstrap resampling with 250 iterations.

All inferred interactions were annotated according to their interaction class in CellChatDB (Secreted Signaling, ECM–receptor, Cell–cell Contact), and for the purposes of the present study we restricted downstream analyses to the Cell–cell Contact category, thereby minimizing the contribution of long-range paracrine signaling. The final analysis produced aggregated interaction probabilities at both group and signaling pathway levels, yielding ranked lists of statistically significant ligand–receptor pairs (*p* < 0.05). Particular attention was given to intracompartment communication patterns for further biological interpretation.

## Results

### Manual spots annotation

**Figure 1:**
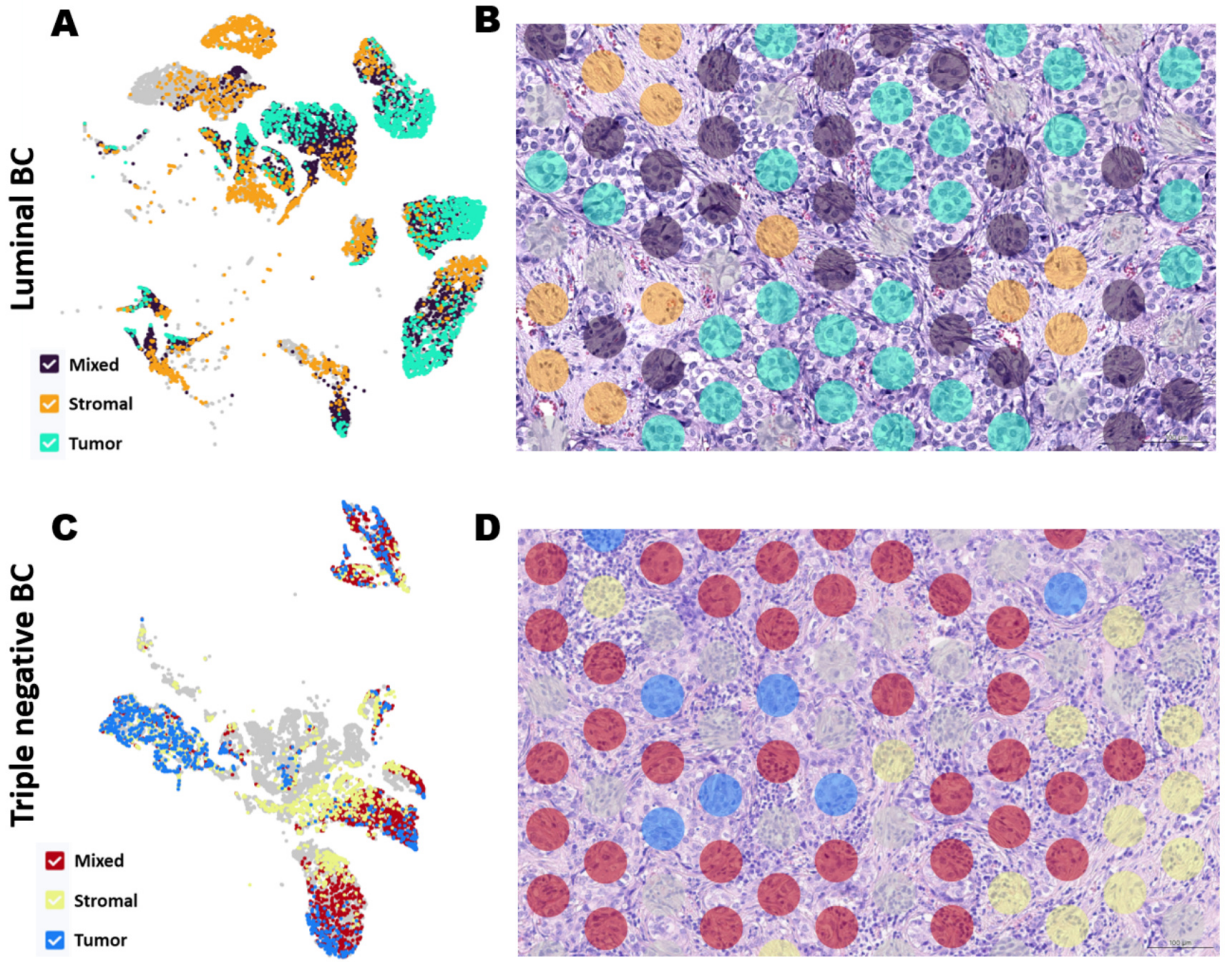
**Classification of spots in the Loupe Browser v8.0.0. A** and **C**: t-SNE; **B** and **D**: manual spots annotation.

Manual morphological annotation of spots was performed directly within the Loupe Browser software v8.0.0 on hematoxylin and eosin-stained tissue by three independent researchers sequentially, ensuring high annotation accuracy. The interobserver discordance rate was below 5%. Discrepancies were resolved by consensus discussion, involving an experienced pathologist when necessary. Spots were classified into three categories: tumor spots (containing tumor cells without contact with stromal cells), stromal spots (containing tumor microenvironment cells not in contact with tumor cells), and mixed spots (containing both tumor cells and stromal cells in contact) (Figure 1).

Classification of spots based on their spatial localization in the tissue was conducted according to the following criteria: tumor spots included only tumor cells and no stromal cells; stromal spots contained only stromal cells of the tumor microenvironment (such as immune cells and fibroblasts) without any tumor cells; mixed spots included both tumor and stromal cells in varying proportions, with each mixed spot comprising at least three tumor cells and at least three stromal cells.

All analyzed tumor tissue regions were localized within the primary tumor mass; transcriptomic features of the invasive tumor margin were outside the scope of this study. Additionally, spots containing no cells, areas with normal mammary gland structures, necrotic regions, vascular structures, erythrocytes, tertiary lymphoid structures, spots situated more than 300 μm from tumor cells, edge spots, as well as spots with artefactual staining or unclear imaging were excluded from analysis.

### DEGs and biological processes

To identify transcriptomic features of tumor and stromal cells in direct contact, we performed the following comparisons: mixed spots versus tumor spots and mixed spots versus stromal spots in luminal BC, as well as mixed spots versus tumor spots and mixed spots versus stromal spots in triple-negative BC. Since mixed spots contain both tumor and stromal cells, both corresponding genes and biological processes are functionally active simultaneously within these regions. In the comparison of mixed spots versus tumor spots, DEGs and biological processes related to tumor growth that are enriched in mixed spots were evaluated. Conversely, in the comparison of mixed spots versus stromal spots, DEGs and processes characteristic of stromal cells—including immune cells, fibroblasts, and endothelial cells—were analyzed.

#### Luminal breast cancer

In luminal BC, the mixed cluster (tumor cells colocalized with stromal cells) showed higher expression of genes encoding S100A family Ca^2+^-binding proteins (*S100A4* (log2FC = 0.61, *p*_adj = 0.0000), *S100A8* (log2FC = 1.99, *p*_adj = 0.0000), *S100A9* (log2FC = 1.88, *p*_adj = 0.0000)), matrix metalloproteinases (*MMP2* (log2FC = 0.64, *p*_adj = 0.0000), *MMP7* (log2FC = 0.79, *p*_adj = 0.0000), *MMP14* (log2FC = 0.59, *p*_adj = 0.0000)), epithelial markers and cytokeratins (*KRT5* (log2FC = 0.70, *p*_adj = 0.0000), *KRT7* (log2FC = 0.68, *p*_adj = 0.0000), *KRT15* (log2FC = 0.93, *p*_adj = 0.0000), *KRT23* (log2FC = 0.67, *p*_adj = 0.0000), *KRT81* (log2FC = 0.75, *p*_adj = 0.0000)), the mesenchymal marker *VIM* (log2FC = 0.65, *p*_adj = 0.0000), and genes associated with epithelial-mesenchymal transition (EMT) (*ICAM1* (log2FC = 0.79, *p*_adj = 0.0000), *PRRX1* (log2FC = 0.82, *p*_adj = 0.0000)) compared to tumor-only cluster.

**Figure 2:**
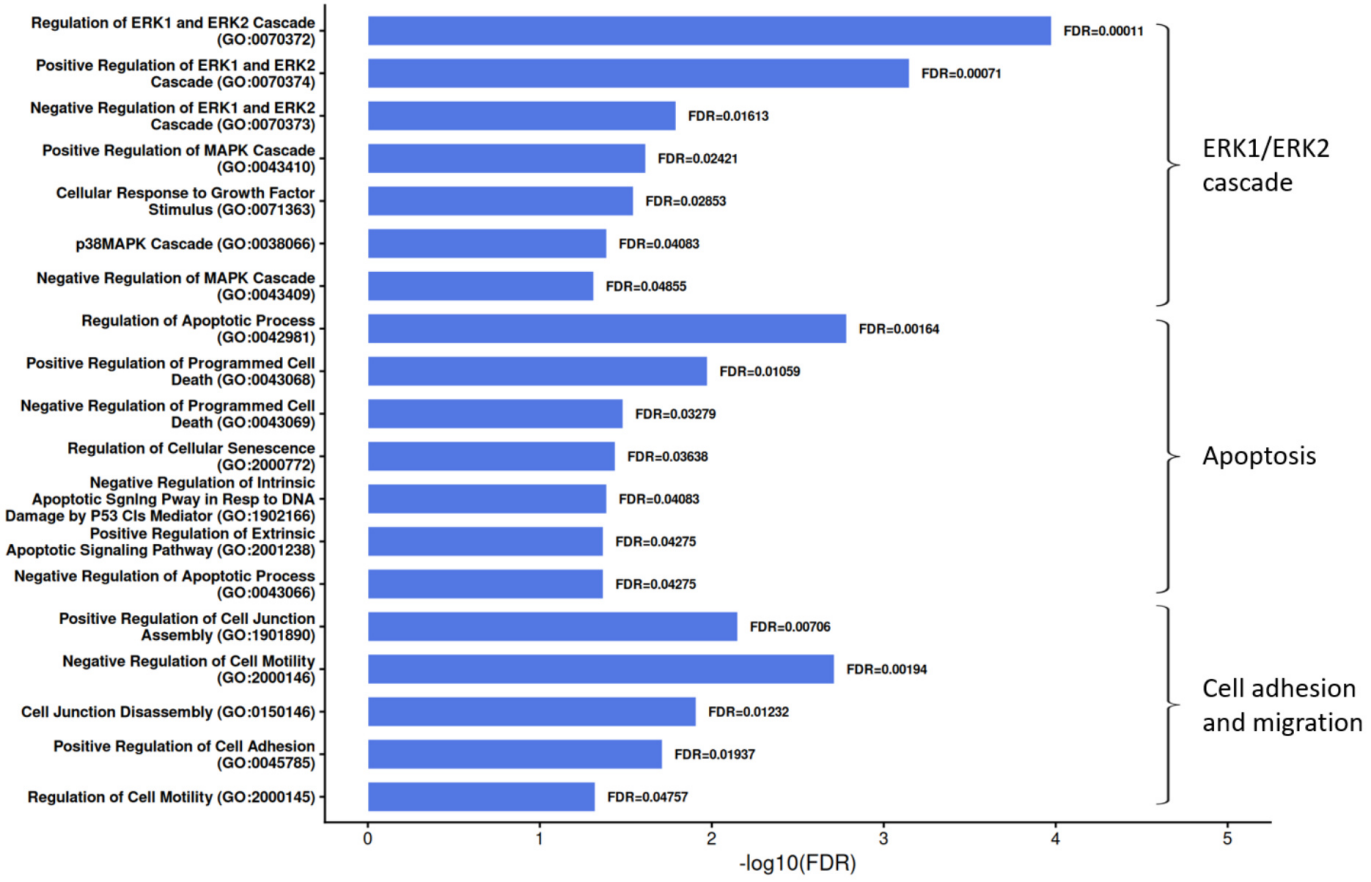
**Biological processes enriched in tumor cells in contact with stromal cells in luminal breast cancer.** FDR: false discovery rate (adjusted *p*-value, Benjamini–Hochberg correction).

Gene Ontology Biological Process analysis for luminal BC revealed that genes upregulated in mixed cluster were enriched for processes related to regulation of the ERK/MAPK cascade (e.g., Regulation of ERK1 and ERK2 Cascade, GO:0070372, *p*_adj = 0.0001; Positive Regulation of ERK1 and ERK2 Cascade, GO:0070374, *p*_adj = 0.0007; Negative Regulation of ERK1 and ERK2 Cascade, GO:0070373, *p*_adj = 0.0161), apoptosis regulation (e.g., Regulation of Apoptotic Process, GO:0042981, *p*_adj = 0.0016; Positive Regulation of Programmed Cell Death, GO:0043068, *p*_adj = 0.0106; Negative Regulation of Programmed Cell Death, GO:0043069, *p*_adj = 0.0328), and regulation of cell adhesion and migration (Positive Regulation of Cell Junction Assembly, GO:1901890, *p*_adj = 0.0071; Negative Regulation of Cell Motility, GO:2000146, *p*_adj = 0.0019; Positive Regulation of Cell Adhesion, GO:0045785, *p*_adj = 0.0194) compared to tumor-only cluster (Figure 2, Table S2).

Stromal cells adjacent to tumor cells (mixed cluster) within the tumor mass in luminal BC exhibited hyperexpression of only two immune response-associated genes, *FDCSP* (log2FC = 1.44, *p*_adj = 0.0000) and *CXCL13* (log2FC = 1.11, *p*_adj = 0.0000), compared to stromal cells neighboring exclusively other stromal cells (stromal cluster), without upregulation of other stromal-related processes. Consistently, enrichment analysis of biological processes did not reveal dominance of immune response, stromal remodeling, angiogenesis, or growth factor production processes in the contact region compared to the stromal areas. All these processes were more active in microenvironmental cells not in direct contact with tumor cells.

#### Triple-negative breast cancer

In triple-negative BC, the mixed cluster showed hyperexpression of S100A family genes *S100A2* (log2FC = 0.97, *p*_adj = 0.0000), *S100A8* (log2FC = 0.60, *p*_adj = 0.0000), and *S100A9* (log2FC = 0.71, *p*_adj = 0.0000), the epithelial gene *KRT6B* (log2FC = 0.73, *p*_adj = 0.0000), the classical cancer stem cell marker *CD44* (log2FC = 0.64, *p*_adj = 0.0000), and the gene *NOTCH2* (log2FC = 0.72, *p*_adj = 0.0000), which is associated with negative regulation of EMT, compared to tumor-only cluster.

**Figure 3:**
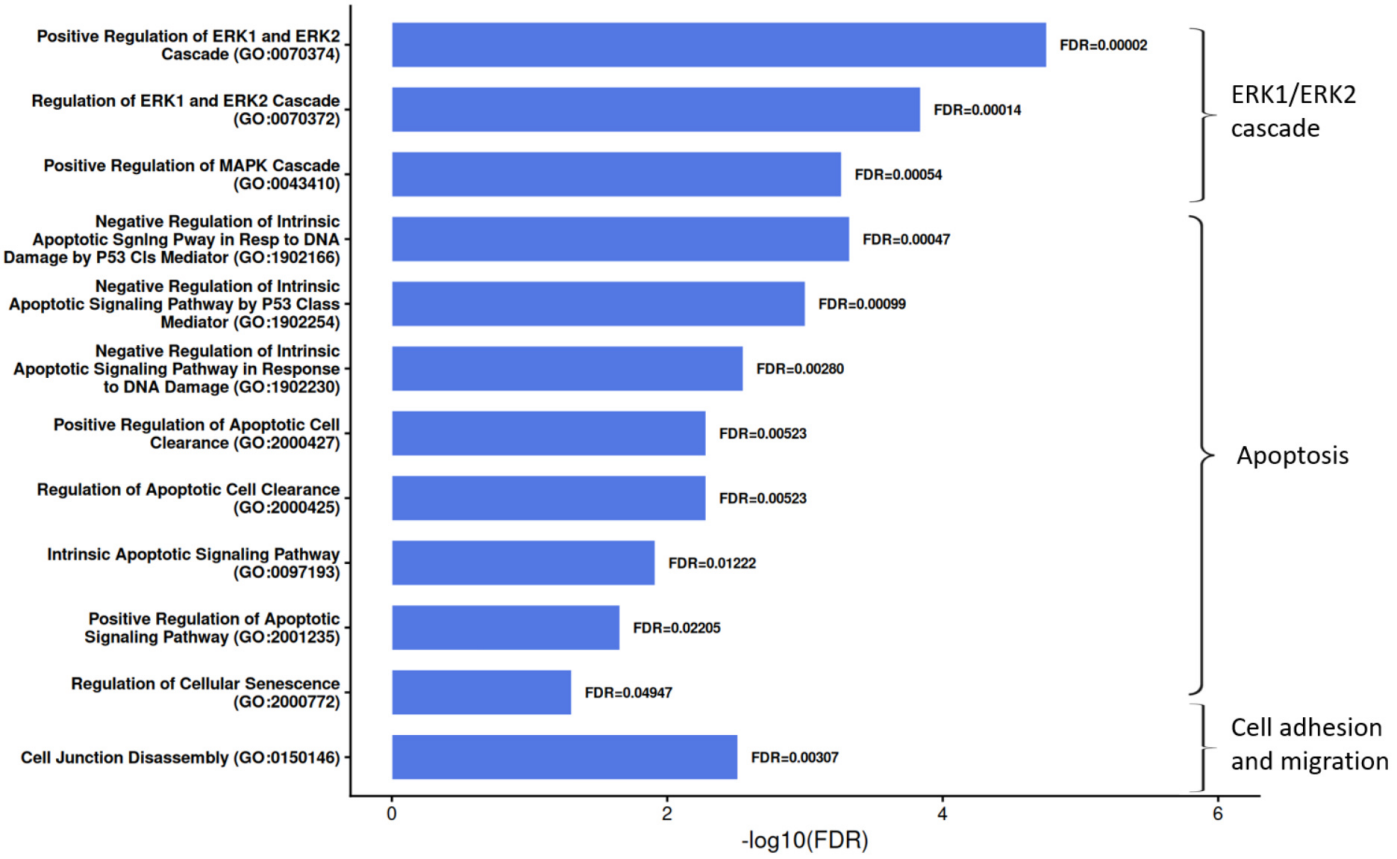
**Biological processes enriched in tumor cells in contact with stromal cells in triple-negative BC.** FDR: false discovery rate (adjusted *p*-value, Benjamini–Hochberg correction).

Similar to luminal BC, mixed cluster (tumor cells colocalized with stromal cells) in triple-negative BC were characterized by increased activity of processes regulating the ERK/MAPK cascade, including Positive Regulation of ERK1 and ERK2 Cascade (GO:0070374, *p*_adj = 0.00002), Regulation of ERK1 and ERK2 Cascade (GO:0070372, *p*_adj = 0.0001), and Positive Regulation of MAPK Cascade (GO:0043410, *p*_adj = 0.0005). Increased regulation was also observed in apoptosis-related pathways, such as Negative Regulation of Intrinsic Apoptotic Signaling Pathway in Response to DNA Damage by p53 Class Mediator (GO:1902166, *p*_adj = 0.0005), Negative Regulation of Intrinsic Apoptotic Signaling Pathway by p53 Class Mediator (GO:1902254, *p*_adj = 0.0010), and Negative Regulation of Intrinsic Apoptotic Signaling Pathway in Response to DNA Damage (GO:1902230, *p*_adj = 0.0028). Additionally, processes involved in cell adhesion and migration, including Cell Junction Disassembly (GO:0150146, *p*_adj = 0.0031), were more active compared to the tumor center (Figure 3, Table S2).

In regions of colocalization between tumor and stromal cells, genes associated with angiogenesis and immune processes, including *VEGFA* (log2FC = 0.86, *p*_adj = 0.0000), *CXCL17* (log2FC = 1.74, *p*_adj = 0.0000), *CCL28* (log2FC = 1.45, *p*_adj = 0.0000), and *HLA-A* (log2FC = 1.04, *p*_adj = 0.0000), were more highly expressed compared to stromal areas distant from the tumor, despite their expression also being detected in tumor cells. Biological process enrichment analysis revealed activation of Regulation of Blood Vessel Branching (GO:1905553, *p*_adj = 0.0233) and Positive Regulation of Leukocyte Chemotaxis (GO:0002690, *p*_adj = 0.0252) in mixed spots; however, similar processes were also activated in stromal spots.

#### Luminal versus triple-negative breast cancer

**Figure 4:**
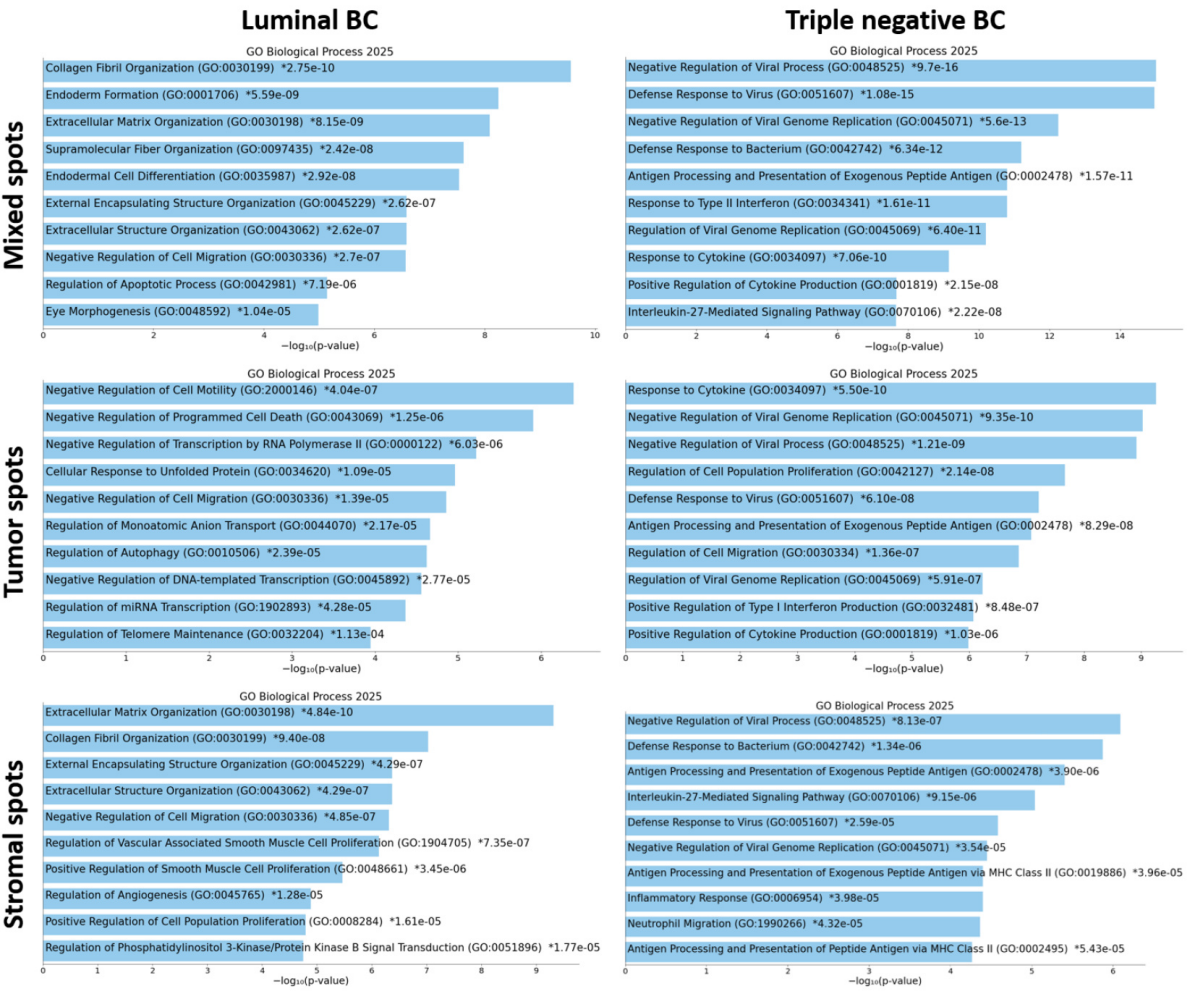
Top 10 enriched biological processes in each cluster in luminal and triple-negative BC (breast cancer).

We also compared transcriptomic features between luminal and triple-negative BС across corresponding spot clusters: mixed spots (luminal BC versus triple-negative BC), stromal spots (luminal BC versus triple-negative BC), and tumor spots (luminal BC versus triple-negative BC). Enriched biological processes were identified for each comparison (Table S2). Consistently, processes related to immune response, inflammation, and cytokine signaling were more highly enriched in all triple-negative BC regions compared to corresponding luminal BC regions. Figure 4 presents the top 10 enriched processes in each region for both studied subtypes.

### Ligand-receptor pairs at the contacted tumor and stromal cells

Using the CellChat tool, we assessed not only ligand-receptor pairs within the colocalization regions of tumor and stromal cells, but also pairs present within individual mixed spots or in neighboring mixed spots (Table 2, Table S3). The maximum distance between detected pairs was 100 µm, while the minimum distance corresponded to immediate cellular neighbors. Table 2 categorizes the ligand-receptor pairs common to both luminal and triple-negative BC, as well as those unique to each subtype. The ligand‑receptor pairs presented in this table are based on the detection of mRNA transcripts encoding the putative ligand and receptor components. These findings indicate the potential for intercellular communication at the protein level but do not constitute direct evidence of ligand‑receptor binding or functional signaling.

**Table 2:** Ligand-receptor pairs in the colocalization regions of tumor and stromal cells in luminal and triple-negative breast cancer (BC): (I) common pairs for luminal and triple-negative BC, (II) pairs unique to luminal BC, (III) pairs unique to triple-negative BC.

**Ligand**	**Receptor**	**Cells expressing ligand**	**Cells expressing receptor**	**Function**	**References**
**I. Luminal and triple-negative BC**					
CDH1	CDH1	tumor cells	tumor cells	epithelial cells adhesion, EMT regulation	[19, 20]
CDH1	ITGA2_ITGB1	tumor cells	tumor cells	cell-cell adhesion	[21]
HLA-DMA	CD4	tumor cells, APCs	T cells	antigen presentation	[22]
HLA-DMB	CD4	tumor cells, APCs	T cells	antigen presentation	[22]
HLA-DQA1	CD4	tumor cells, APCs	T cells	antigen presentation	[22]
OCLN	OCLN	tumor cells	tumor cells	tight junctions	[23]
**II. Luminal BC**					
APP	CD74	tumor cells	macrophages	suppressing the phagocytic activity of macrophages	[24]
CD46	JAG1	T cells	epithelial cells, endothelial cells	induction of T helper 1 effector T cells and their switching into regulatory T cells	[25, 26]
CD99	CD99L2	tumor cells, immune cells	tumor cells, immune cells	adhesion, transendothelial migration, differentiation, cell death	[27]
CDH5	CDH5	tumor cells, endothelial cells	tumor cells, endothelial cells	epithelial cells, endothelial cells adhesion	[28]
DSC2	DSG2	tumor cells	tumor cells	formation of desmosomes	[29]
DSG2	DSC3	tumor cells	tumor cells	formation of desmosomes	[29]
ENTPD1	ADORA1	tumor cells, endothelial cells, immune cells	immune cells	activating immune suppressor cells	[30]
HLA-DPA1	CD4	tumor cells, APCs	T cells	antigen presentation	[22]
HLA-DPB1	CD4	tumor cells, APCs	T cells	antigen presentation	[22]
HLA-DRA	CD4	tumor cells, APCs	T cells	antigen presentation	[22]
HLA-DRB1	CD4	tumor cells, APCs	T cells	antigen presentation	[22]
HLA-DRB5	CD4	tumor cells, APCs	T cells	antigen presentation	[22]
HLA-DOA	CD4	tumor cells, APCs	T cells	antigen presentation	[22]
ICAM1	ITGAX_ITGB2	tumor cells, endothelial cells, immune cells	immune cells	stabilizing cell-cell interactions and promoting leukocyte-endothelial migration	[31]
ICAM1	ITGAM_ITGB2	tumor cells, endothelial cells, immune cells	immune cells	stabilizing cell-cell interactions and promoting leukocyte-endothelial migration	[31]
ITGA9_ITGB1	VCAM1	tumor cells	endothelial cells, tumor cells	cell adhesion, migration	[32, 33]
ITGA4_ITGB7	VCAM1	leukocytes	endothelial cells, tumor cells	rolling and firm adhesion	[32]
ITGA4_ITGB1	VCAM1	leukocytes	endothelial cells, tumor cells	rolling and firm adhesion	[32]
ITGB2	ICAM1	leukocytes	tumor cells, endothelial cells, immune cells	leukocyte trafficking and adhesion	[34]
JAM2	ITGAV_ITGB1	tumor cells, endothelial cells, leukocytes	immune cells	leukocyte trafficking	[35]
JAM2	ITGA3_ITGB1	tumor cells, endothelial cells, leukocytes	immune cells	leukocyte trafficking	[35]
JAM3	ITGAM_ITGB2	tumor cells, endothelial cells, leukocytes	immune cells	leukocyte trafficking	[36]
JAM2	JAM2	tumor cells, endothelial cells, leukocytes	tumor cells, endothelial cells, leukocytes	tight junctions, leukocyte trafficking	[35]
JAM2	JAM3	tumor cells, endothelial cells, leukocytes	tumor cells, endothelial cells, leukocytes	tight junctions, leukocyte trafficking	[37]
JAM3	JAM2	tumor cells, endothelial cells, leukocytes	tumor cells, endothelial cells, leukocytes	tight junctions, leukocyte trafficking	[37]
JAM3	JAM3	tumor cells, endothelial cells, leukocytes	tumor cells, endothelial cells, leukocytes	tight junctions, leukocyte trafficking	[37]
PECAM1	PECAM1	endothelial cells, tumor cells	endothelial cells, tumor cells	angiogenesis, maintenance of endothelial barrier integrity	[38]
THY1	ITGAV_ITGB3	endothelial cells, fibroblasts	tumor cells	adhesion and transmigration of tumor cells	[39]
THY1	ITGAX_ITGB2	endothelial cells, fibroblasts	immune cells	leukocyte adhesion to activated endothelium	[40]
THY1	ITGAM_ITGB2	endothelial cells, fibroblasts	immune cells	leukocyte adhesion to activated endothelium	[40]
**III. Triple-negative BC**					
ADGRE5	CD55	tumor cells, immune cells	tumor cells, immune cells	modulation of various tumorigenic mechanisms	[41]
F11R	F11R	tumor cells, endothelial cells, leukocytes	tumor cells, endothelial cells, leukocytes	tight junctions, EMT regulation, leukocyte trafficking	[42]
F11R	ITGAL_ITGB2	tumor cells, endothelial cells, leukocytes	immune cells	leukocyte transendothelial migration	[36]

In both BC subtypes, various ligand-receptor pairs were identified that are involved in tight junctions, epithelial cell adhesion, cell-cell adhesion, EMT regulation, leukocyte trafficking, leukocyte transendothelial migration, induction of suppressive tumor-associated macrophages (TAMs), and antigen presentation. The list of unique ligand-receptor pairs detected in luminal BC was substantially larger than that in triple-negative BC.

In addition to the processes mentioned above, luminal BC exhibited ligand–receptor pairs associated with formation of desmosomes, cell adhesion to the extracellular matrix and cell–cell communication, tumor and endothelial cell migration, adhesion and transmigration of tumor cells through endothelial cells, angiogenesis, maintenance of endothelial barrier integrity, stabilization of cell–cell interactions, promotion of leukocyte–endothelial migration, leukocyte adhesion to activated endothelium including rolling and firm adhesion, suppression of macrophage phagocytic activity, and activation of immunosuppressive cells.

In contrast, only three unique ligand-receptor pairs, *ADGRE5-CD55*, *F11R-F11R*, *F11R-(ITGAL_ITGB2)*, were identified in triple-negative BC, which play roles in modulation of various tumorigenic mechanisms, tight junctions, EMT regulation and leukocyte trafficking.

## Discussion

Active interaction between tumor cells and stromal components (fibroblasts, immune cells, endothelium) is a driving force in tumor progression. In the study by J. Kaufmann et al. [43], a prognostic parameter called the Tumor-Stroma Contact Ratio was proposed for patients with oropharyngeal cancer. This parameter evaluates the relationship between tumor cells in contact with the stromal surface and the total number of epithelial tumor cells and predicts poor response to (chemo-)radiotherapy, as well as shorter overall and progression-free survival in locally advanced oropharyngeal cancer [43]. This work highlights the particular importance of investigating the characteristics of the direct contact zone between tumor nests and stroma, where the most intense intercellular communication occurs.

In our study, we employed manual morphological annotation of Visium 10x tumor tissue spots, selectively highlighting regions of interest and excluding non-representative areas. This approach provided preliminary evidence that tumor cells in contact with stromal cells in luminal BC show increased expression of genes including *S100A4*, *S100A8*, *S100A9*, *MMP2*, *MMP7*, *MMP14*, *KRT5*, *KRT7*, *KRT15*, *KRT23*, *KRT81*, *VIM*, *ICAM1*, and *PRRX1*. In triple-negative BC, the contacting tumor cells showed increased expression of *S100A2*, *S100A8*, *S100A9*, *KRT6B*, *CD44*, and *NOTCH2*.

The functional versatility of S100 proteins derives from their ability to interact with numerous targets, enabling their involvement in critical processes such as Ca^2+^ signaling, cell growth, differentiation, apoptosis, inflammation, and motility [44]. Data regarding their prognostic role are contradictory [44], but their detection at the tumor boundary may indicate increased intra- and intercellular interaction activity.

MMPs play a critical role in cancer metastasis by degrading the extracellular matrix and blood vessel walls, enabling tumor cells to invade and spread to distant organs [45]. In luminal BC, higher expression of MMP genes was observed in tumor cells at the tumor-stroma edge, whereas in triple-negative BC, no differences in MMP gene expression were found between mixed and tumor clusters. This may indicate that both edge and central tumor cells are equally adapted to interact with and degrade the extracellular matrix, potentially contributing to the aggressiveness of this subtype. Comparison of tumor-stromal co-localization regions between luminal and triple-negative BC showed a relatively higher enrichment of immune response‑associated GO terms in triple‑negative BC, including those related to innate immunity, inflammation, immune cell recruitment, secretion, and cytokine signaling. Notably, these processes were even more highly enriched in triple-negative BC stromal regions not in contact with tumor cells and in tumor regions not in contact with stroma compared to luminal BC. Therefore, the enrichment of immune-related processes in triple-negative BC characterizes not only the tumor-immune cell interface but also the entire tumor mass. This indicates that heightened immune activity results not solely from direct tumor-immune cell contact but also from active paracrine interactions between spatially separated cell populations.

Interestingly, we detected hyperexpression of the *CD44* gene in tumor cells at the tumor-stroma interface in triple-negative BC. CD44 is strongly linked to cancer stem cell hallmarks, notably self-renewal, EMT, therapy resistance, and enhanced tumorigenicity. In the study by O. Mansour et al. [46], CD44 expression showed significant positive correlations with BRCA1, p38 MAPK, E-cadherin, DSG1, HOTAIR, and BC200, suggesting its involvement in both stemness and tumor progression pathways in BC [46]. Expression of stemness markers in the CD44^hi^/CD24^low^ and ALDH^hi^ combination in tumors of BC patients correlates with shorter disease-free survival and overall survival in those receiving neoadjuvant chemotherapy [47]. The elevated expression of CD44 at the tumor-stroma interface observed in our study underscores its pro-tumorigenic functional significance in BС. However, it has also been shown that ALDH1^+^ cancer stem cells demonstrate superior self-renewal and tumorigenic abilities compared to CD44^hi^/CD24^low/–^ cancer stem cells across different molecular subtypes of BC [48]. Differences in the expression patterns of cytokeratin genes and EMT-associated genes might reflect fundamentally distinct strategies of invasion and cellular plasticity between BC subtypes.

In both BC subtypes, the set of genes upregulated in tumor cells colocalized with stromal cells was enriched for GO terms related to regulation of the ERK/MAPK cascade, apoptosis, cell adhesion, and migration. The ERK/MAPK signaling pathway is closely associated with cell proliferation and differentiation and plays a pivotal role in the cellular signal transduction network. It also participates in extracellular matrix degradation by upregulating MMPs expression, thereby promoting tumor invasion and metastasis [49]. It is also known that ERK1/2 can exert pro-apoptotic effects [50], whereas the JNK and p38 MAPK cascades mediate pro-apoptotic processes [51].

Apoptosis is recognized as a process with “double-edged” consequences: on one hand, it is tumor-suppressive by eliminating malignant or pre-malignant cells; on the other hand, it promotes tumor progression by stimulating reparative and regenerative responses in the tumor microenvironment [52]. The enrichment of these processes in co-localized regions reflects not only the high invasive potential of tumor cells in these areas but also the intensified cellular response to growth-restrictive conditions in the surrounding microenvironment. This phenomenon is characteristic of both luminal and triple-negative BC. On the other hand, activation of these pathways in tumor cells may be induced by factors secreted by stromal cells. For example, the tumor microenvironment can activate the JAK/STAT pathway through factors such as IL-6, which synergizes with ERK signaling to further diminish the efficacy of targeted therapies [53]. Additionally, induction of the extrinsic apoptosis pathway in tumor cells requires interaction between death receptors and their corresponding ligands (TNF-α, FasL, and TRAIL), which are predominantly expressed in immune cells, including granulocytes, monocytes, T cells, B cells, dendritic cells, and NK cells [54].

In regions of tumor-stromal cell contact in luminal BС, despite its characterization as an immunologically cold tumor, a broader spectrum of genes encoding various HLA class II molecules co-expressed with the *CD4* gene was observed compared to triple-negative BC. Our data corroborate findings obtained using a different approach by the research group led by Y. Cui et al. [55], which identified a distinct tumor subpopulation that upregulates MHC-II genes and actively interacts with immune cells in triple-negative BC [55]. According to recent studies, effective antitumor immunity in BC may be linked to MHC-II-restricted presentation of optimal tumor antigens and requires the cooperation of CD4^+^ T cells, B cells, and antigen-specific antibodies [56]. In experimental models, MHC-II-restricted neoantigen vaccination enhances inflammatory signaling within the tumor microenvironment of “cold” tumors, increases infiltration of CD4^+^ and CD8^+^ T cells and IFN-γ production, and exhibits synergy with immune checkpoint inhibitors [57]. Given that luminal BC subtypes are classified as immunologically “cold” tumors, this strategy holds promise for their treatment. In this context, our observed enrichment of HLA-II/CD4 pairs in areas of direct contact between tumor and stromal cells suggests that these spatial niches may serve as potential targets for MHC-II-directed immunotherapy, particularly in patients with luminal BC.

Other notable immune cell interactions detected include the APP–CD74 axis. APP expressed on tumor cells binds to CD74 on TAMs, delivering an inhibitory signal that suppresses TAMs phagocytic activity [24]. This mechanism enables tumor cells to evade immune-mediated destruction, thereby promoting tumor progression. In a study by H. Zeng et al. [58], a ligand‑receptor pair‑based signature score derived from on‑treatment tumor specimens was developed to predict immune checkpoint blockade response in metastatic melanoma. This score included seven ligand‑receptor pairs, among them APP–CD74 [58]. Another study by O. Chen et al. [59] demonstrated that modulating tumor‑associated macrophages through APP–CD74 blockade using IL4R‑exosomes synergizes with PD‑1 inhibition in gastric cancer mouse models [59]. In BC, the prognostic or therapeutic significance of this axis has not been established and requires validation.

In our study, tumor-stroma co-localization regions in luminal BC exhibited a greater number of ligand-receptor pairs involved in tight junction formation between tumor cells and immune cell recruitment to the tumor compared to triple-negative BC. Such interactions may restrict tumor growth and enhance anti-tumor immune responses. Notably, a broad range of these ligand-receptor pairs included integrins composed of various α- and β-subunits. Integrin expression and activity are closely associated with multiple stages of tumor development, such as initiation, angiogenesis, motility, invasion, and metastasis. While some integrins promote tumor formation or potentiate oncogenic signaling through receptor interactions, others have minimal or even inhibitory effects [60]. Previous studies have investigated the spectrum of integrin interactions with other molecules, demonstrating their high heterogeneity in BC [12]. The participation of integrins in a wide range of molecular partnerships likely underlies their functional duality. Anti‑integrin therapy is currently used in the treatment of ulcerative colitis and Crohn’s disease [61]. Accumulating evidence also supports the potential of anti‑integrin approaches in cancer. For instance, inhibition of integrin αVβ3 has been shown to induce cytotoxicity and suppress migration in ovarian cancer cells [62]. Moreover, integrin‑targeting molecules may be employed to improve the efficiency of targeted drug delivery to tumors [63].

Also, we identified an expanded repertoire of JAM family interactions in luminal BC, including homotypic (*JAM2-JAM2*, *JAM3-JAM3*) and heterotypic (*JAM2-JAM3*) contacts as well as integrin-mediated pairs (*JAM2-ITGAV_ITGB1*, *JAM2-ITGA3_ITGB1*, *JAM3-ITGAM_ITGB2*). Given that JAM3 acts as a tumor suppressor in BC brain metastases [37] and JAM2 is involved in leukocyte trafficking and angiogenesis [35], the enrichment of these contacts specifically at the tumor-stroma interface suggests they may contribute to the less aggressive phenotype of luminal BC.

Our study aligns with the rapidly expanding field of spatial transcriptomics in BC, an approach that holds promise for refining the molecular classification of this disease and identifying spatial tumor microenvironment structures relevant to the development of personalized therapy [64, 65]. A key advantage of spatial approaches is that they preserve tissue architecture, which is critically important for investigating interactions between tumor and stromal cells. Currently, few studies comprehensively investigate the characteristics of tumor and stromal cells in direct contact. For example, Wu et al. [8] demonstrated that the tumor-stroma edge is characterized by extensive extracellular matrix remodeling, immunomodulatory regulation, and EMT. They also identified significant interactions between cancer-associated fibroblasts (CAFs) and M2-like TAMs that contribute to immune exclusion and drug resistance [8]. Additionally, the spatial organization and immune status of the tumor-stroma edge were identified as distinctive features differentiating mismatch repair-deficient (dMMR) and proficient (pMMR) colorectal cancers, correlating with responses to immune checkpoint blockade therapy [66]. Importantly, these studies investigated the invasive front—the tumor edge with adjacent normal tissue. In contrast, our study focused on the characteristics of tumor and stromal cells located within the tumor mass itself. Our approach is consistent with existing clinical guidelines for the assessment of TILs in BC, which restrict analysis to the stroma within the invasive margin of the tumor rather than the invasive edge with adjacent normal tissue [67]. It is within these internal tumor regions that therapy-resistant tumor cell populations and progression-driving clones emerge, alongside high concentrations of immunosuppressive cells. Thus, studies of tumor and stromal cells in direct contact within the tumor nodules may provide refined insights into mechanisms of tumor progression and identify novel therapeutic targets for malignant neoplasms.

The main limitation of our study is the cohort size—19 patients (10 with luminal BC and 9 with TNBC). On the one hand, this number is comparable to many pilot spatial transcriptomics studies, and the large number of spatial spots analyzed per sample provides adequate statistical power. On the other hand, the high interpatient heterogeneity of BC, particularly in the triple-negative BC group, raises the possibility that some variants of gene expression or ligand-receptor pairs may have remained undetected. Therefore, the presented results should be considered hypothesis‑generating; independent validation cohorts using additional analytical methods (multiplex immunohistochemistry, spatial sequencing on other platforms) are required to confirm these findings.

## Abbreviations

BC: breast cancer
DEGs: differentially expressed genes
EGFR: epidermal growth factor receptor
EMT: epithelial-mesenchymal transition
ER: estrogen receptor
FFPE: formalin-fixed, paraffin-embedded
HER2: human epidermal growth factor receptor 2
PR: progesterone receptor
TAMs: tumor-associated macrophages
TILs: tumor-infiltrating lymphocytes

## Supplementary materials

The supplementary Table S1 for this article is available at: https://www.explorationpub.com/uploads/Article/file/1002388_sup_1.xlsx. The supplementary Table S2 for this article is available at: https://www.explorationpub.com/uploads/Article/file/1002388_sup_2.xlsx. The supplementary Table S3 for this article is available at: https://www.explorationpub.com/uploads/Article/file/1002388_sup_3.xlsx.

## Declarations

### Availability of data and materials

Breast cancer spatial transcriptomics sequencing data used in this publication are deposited in the NCBI Gene Expression Omnibus (GEO) database and available through GEO Series accession number GSE242311 (https://www.ncbi.nlm.nih.gov/geo/query/acc.cgi?acc=GSE242311). Processed data supporting the findings are available from the corresponding author upon reasonable request.

### Copyright

© The Author(s) 2026.
